# Microbiome-Augmented Model for Predicting Knee Osteoarthritis Progression Based on Gut Microbiota and Kellgren-Lawrence Classification

**DOI:** 10.7759/cureus.73402

**Published:** 2024-11-10

**Authors:** Lei Jiang, Shankai Liu, Hongyang Kong

**Affiliations:** 1 Department of Orthopedics, The Affiliated Taizhou People's Hospital of Nanjing Medical University, Taizhou, CHN; 2 Department of Interventional Radiology, The Affiliated Taizhou People's Hospital of Nanjing Medical University, Taizhou, CHN

**Keywords:** gut microbiota, kellgren-lawrence classification, osteoarthritis, prediction model, progression

## Abstract

Purpose: Knee osteoarthritis (OA) is a widespread chronic degenerative condition that may experience slow or rapid deterioration. The gut-joint axis represents a bidirectional relationship in OA onset and progression. This study aimed to establish and validate a prediction model of knee OA disease progression.

Methods: This prospective cohort investigation involved 296 patients diagnosed with knee OA using X-ray and CT scans at Taizhou People’s Hospital from January 2020 to January 2022. Fecal samples and general information were collected for gut microbiota analysis. Least absolute shrinkage and selection operator (LASSO) regression and various prediction models, including microbiome-augmented models, were employed for knee OA risk prediction. The models predicting Kellgren-Lawrence classification one year later were evaluated by accuracy, sensitivity, specificity, positive predictive value (PPV), negative predictive value (NPV), and area under the curve (AUC).

Results: A total of 270 patients were involved in our study. After random assignment, 214 patients belonged to the training set and 56 patients belonged to the test set. The final intestinal flora included in the analysis included the following 12 species. Shannon index of patients with a Grade I Kellgren-Lawrence Classification after one year was lower than those with a Grade II/III after one year (P=0.018). The best model was the microbiome-augmented model built by Light GBM (LGBM). The AUC of this model in the training set was 0.812 (0.754-0.870), the sensitivity was 0.804 (0.725-0.883), the specificity was 0.744 (0.664-0.823), the PPV was 0.722 (0.638-0.807), the NPV was 0.821 (0.748-0.894), and the accuracy was 0.771 (0.715-0.827). The AUC of this model in the testing set was 0.876 (0.781-0.972), the sensitivity was 0.759 (0.603-0.914), the specificity was 0.917 (0.806-1.000), the PPV was 0.917 (0.806-1.000), the NPV was 0.759 (0.603-0.914), and the accuracy was 0.830 (0.729-0.931).

Conclusion: One year later, the microbiome-augmented model constructed by LGBM for knee OA patients based on general and gut microbiota data using the Kellgren-Lawrence classification demonstrated the highest performance. This approach could aid in identifying patients at risk of rapid disease progression, facilitating early intervention and personalized treatments. Furthermore, it offers a novel perspective on the gut-joint axis's role in OA.

## Introduction

Knee osteoarthritis (OA) is a prevalent chronic degenerative condition that primarily affects the articular cartilage of middle-aged and elderly individuals. It is characterized by the progressive deterioration of cartilage, subchondral sclerosis, hyperplasia, periarticular osteophyte formation, and synovial lesions. These changes can result in joint pain and dysfunction, and potentially lead to joint deformity or disability [1,2]. As the global population ages, OA has become a major public health concern worldwide and a leading cause of medical expenses for middle-aged and older adults. By 2020, OA had become the fourth most common disease globally [3-5].

However, the traditional view of OA as a slow-progressing disease has been challenged, as recent studies have demonstrated that the progression and severity of OA can vary significantly among individuals, with some experiencing rapid deterioration [6-9]. Unfortunately, current diagnostic methods often detect OA at moderate stages, when preventive measures are more difficult to implement effectively. Existing approaches rely on demographic and clinical parameters, sometimes supplemented by radiography, but they often lack the specificity and sensitivity needed for an early and accurate diagnosis [10].

The gut microbiome, a complex ecosystem of microorganisms residing in the intestinal tract, plays a critical role in maintaining overall health. It is involved in nutrient metabolism, immune system regulation, and the maintenance of the intestinal barrier. The gut-joint axis represents a bidirectional relationship, with gut microbiota influencing OA development by modulating immune responses and inflammation. The gut-joint axis refers to the mutual communication signals between the intestinal microbiome and the skeletal joint system, which may mediate bone remodeling, joint inflammation, and other abnormalities. Dysbiosis of the gut microbiome can increase intestinal permeability, allowing bacterial products to translocate into circulation, leading to systemic inflammation and contributing to OA development [11,12]. The gut microbiome offers a novel and non-invasive approach to predicting knee OA progression. Recent studies have identified specific gut microbiota alterations associated with knee OA severity and clinical symptoms, suggesting its potential as a biomarker for disease progression. Consequently, there is a growing interest in evaluating the gut microbiome to better understand and predict OA progression.

As a prospective cohort study, this research involved patients with knee OA. By gathering both general information and gut microbiota data from these patients, the study aimed to explore the role of the gut-joint axis in the development of knee OA and assess its predictive value by establishing and validating a model for predicting the disease's progression.

## Materials and methods

Study population

**Figure 1:**
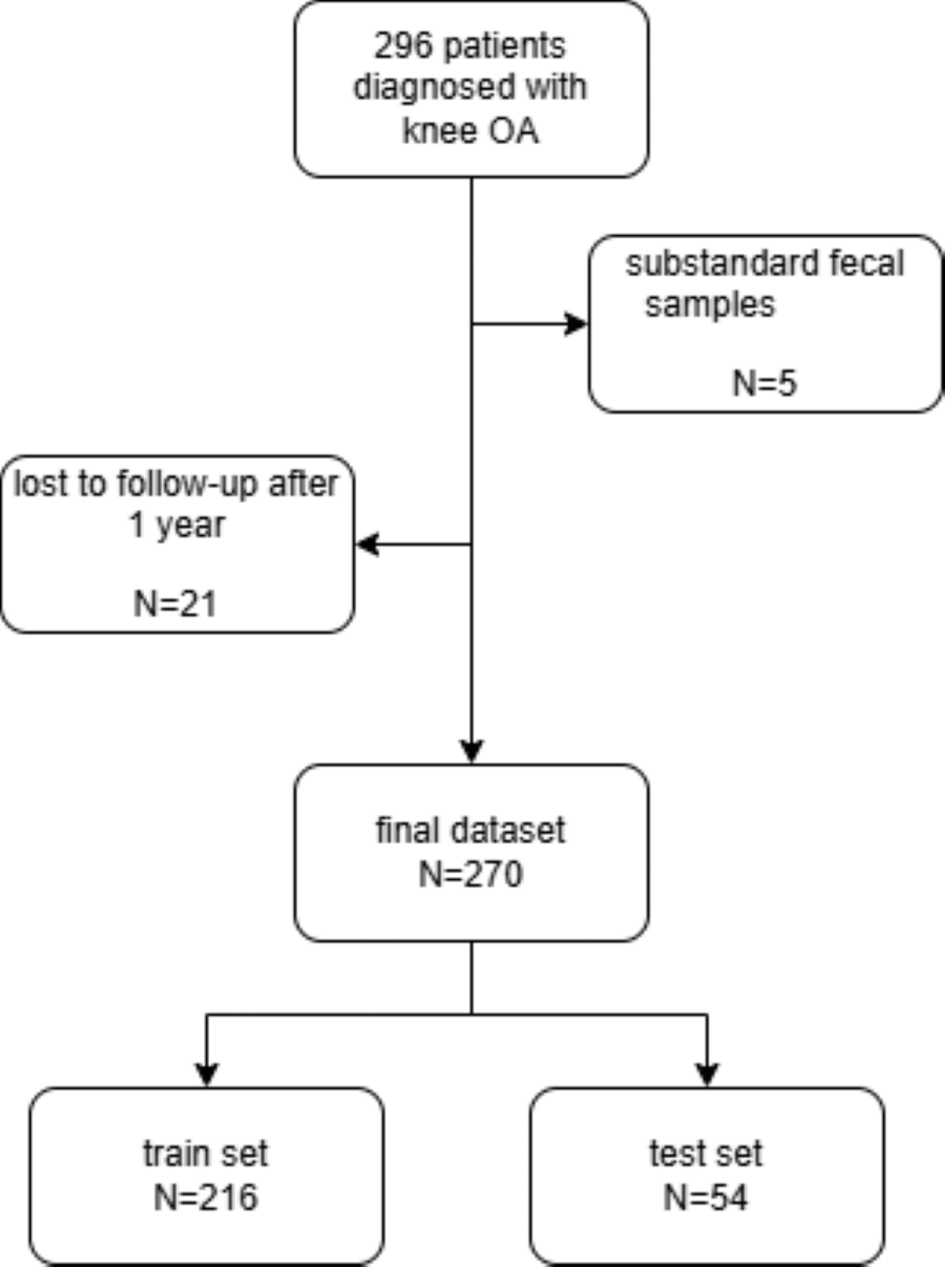
Detailed inclusion and exclusion of study subjects OA, osteoarthritis.

This study was a prospective cohort study that included 296 patients diagnosed with knee OA via X-ray and CT at Taizhou People’s Hospital between January 2020 and January 2022. The inclusion criteria were 1) age between 40 and 80 years; 2) diagnosis of knee OA (Grade I) according to the Kellgren & Lawrence classification (Grade 0 [normal knee], Grade I, Grade II, Grade III, and Grade IV). Five subjects were excluded due to substandard fecal samples, and 21 participants were excluded for being lost to follow-up after one year. Subjects were randomly assigned to either the training set or the test set in an 8:2 ratio. Detailed inclusion and exclusion criteria for the study subjects are shown in Figure 1. The statistical certainty calculated by PASS 15.0.5 (NCSS, LLC., Kaysville, UT) was greater than 0.99.

Variables

General information, including age, gender, body mass index (BMI), and laboratory test results such as fasting blood glucose (FBG), white blood cell count (WBC), and C-reactive protein (CRP), as well as the use of non-steroidal anti-inflammatory drugs (NSAIDs) and knee morphology, were collected. An orthopedic surgeon assessed the patients' knee symptoms at the beginning of the study and after six weeks using the Western Ontario and McMaster Universities Osteoarthritis Index (WOMAC). This index consists of 24 items divided into three categories: pain (five items), stiffness (two items), and physical function (17 items). Each question is rated on a scale from 0 to 4, ranging from none (0) to extreme (4). The scores for each category are summed, yielding a possible range of 0-20 for pain, 0-8 for stiffness, and 0-68 for physical function. The total WOMAC score is obtained by combining all three category scores. The Kellgren-Lawrence classification was used to assess the severity of OA in patients [13]. Two orthopedic surgeons with more than five years of experience determined the Kellgren-Lawrence classification. One year after enrollment, the Kellgren-Lawrence classification was used again to evaluate the patients' outcomes, focusing on Grade I and Grade II/III.

Collection and processing of fecal samples and acquisition of gut microbiota data

In the study, patients were provided with instructions and sampling tools for collecting fresh stool samples. The tools included sampling spoons, gloves, paper toilet covers, and stool collection tubes containing 5 ml of 95% alcohol and small glass beads. Patients were instructed to take a spoonful of the sample, about the size of a peanut, from the middle of the feces using the sampling spoon. They then placed the spoon with the specimen into the stool collection tube, tightened it, and shook it 10 times to mix the stool with the alcohol. Finally, the stool collection tube was securely tightened into an external protection tube. Both patients and samples underwent consistency and quality control checks. The collection personnel delivered the samples to the laboratory for processing on the same day. After mixing, the samples were divided into two equal portions and stored in a cryogenic refrigerator at -80°C. DNA of the intestinal flora was extracted using the magnetic beads method and the fecal genome extraction kit (Ace111) from Shanghai Jingxiang Biotechnology Co., Ltd., Shanghai. The Roche LightCycler 480II (Roche Holding, Basel, Switzerland) was used to amplify the V4 hypervariable region of 16S rDNA of the extracted gut microbiota DNA via PCR. The upstream primer sequence was 5'-GAGTGCCAGCMGCCGCGGTAA-3' and the downstream primer sequence was 5'-ACGGACTACHVGGGTWTCTAAT-3'. Sequencing was performed using Illumina HiSeq X10 (Illumina, Inc., San Diego, CA), targeting the V4 hypervariable region of 16S rDNA. Finally, the Shannon index, Simpson index, and Chao1 index of gut microbiota at the genus level were analyzed.

Statistical analysis

The Shapiro-Wilk test was used to assess the normality of the measurement data. For continuous variables following a normal distribution, results were expressed as mean ± standard deviation (mean±SD), and comparisons between groups were conducted using a t-test. For measurement data that did not follow a normal distribution, values were presented as the median (Q1, Q3), and group comparisons were performed using the Mann-Whitney U test. Categorical data were presented as n (%), and comparisons between groups were made using either the chi-square test or Fisher's exact test. All samples were randomly divided into a training set (n=214) and a testing set (n=56) with an 8:2 ratio. Feature selection was performed using the least absolute shrinkage and selection operator (LASSO) regression with five-fold cross-validation to identify optimal predictors for the model. Logistic regression, Random Forest, XGBoost, and Light GBM (LGBM) models were used to establish prediction models, which were evaluated based on accuracy, sensitivity, specificity, positive predictive value (PPV), negative predictive value (NPV), and area under the curve (AUC). AUC differences were compared using the Delong test. Initially, a conventional risk factor model was established using age, BMI, WOMAC, FBG, and CRP. Then, a microbiome model was developed using gut microbiota indicators. Finally, microbiome-augmented models were created by combining all variables. The models were evaluated through calibration analysis and decision curve analysis. The model with the best performance was selected as the final prediction model. Statistical analyses were performed using R software (R version 4.3.3, 2024-02-29 ucrt; R Foundation for Statistical Computing, Vienna, Austria), with all tests conducted as two-sided and P<0.05 considered statistically significant.

## Results

Gut microbiota abundance after screening

**Figure 2:**
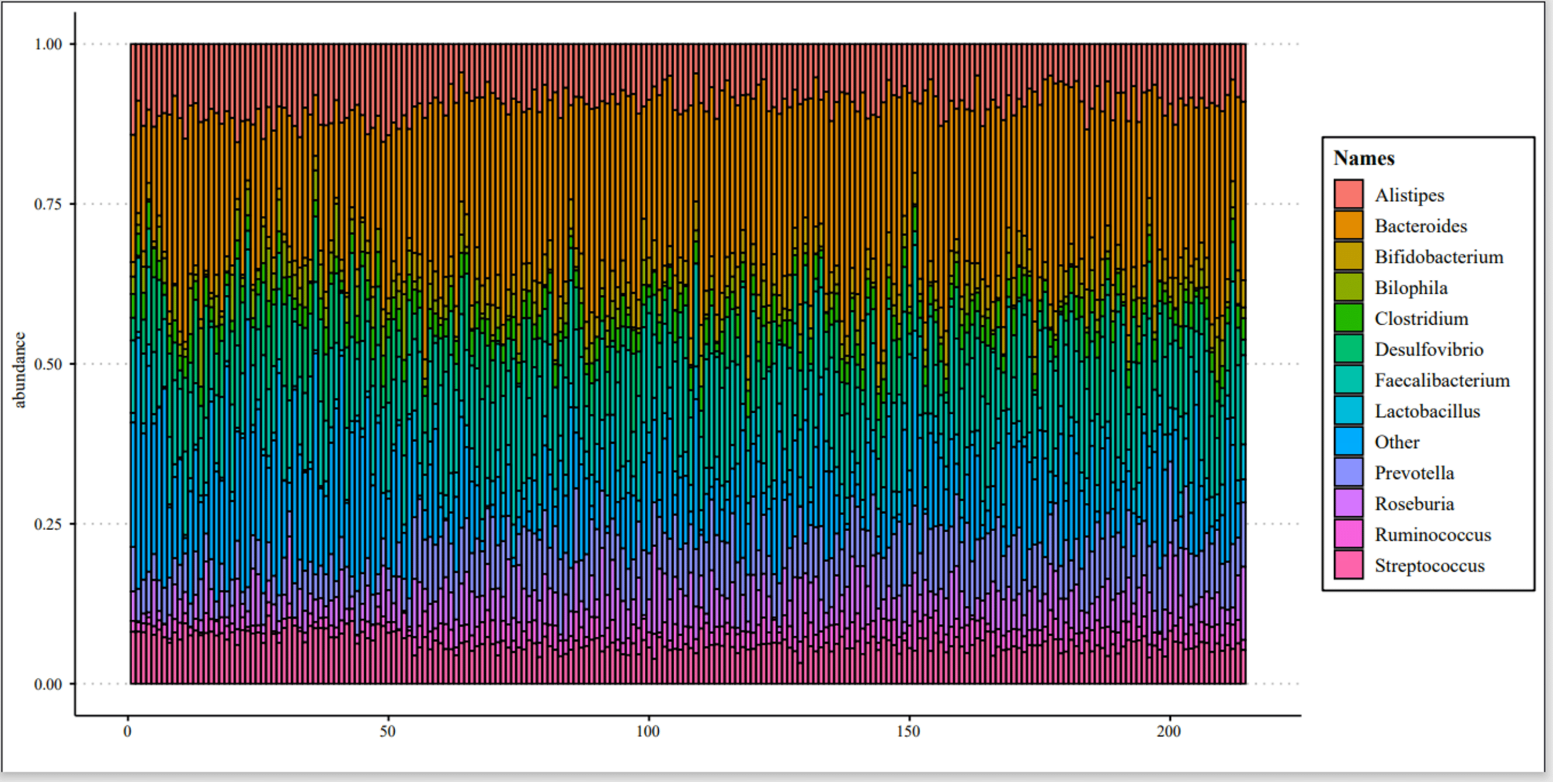
Relative abundance data of 12 screened intestinal flora

LASSO regression with five-fold cross-validation was applied to screen the genus-level abundance data of all intestinal flora. The final analysis included 12 bacterial species: *Bacteroides*, *Prevotella*, *Lactobacillus*, *Bilophila*, *Desulfovibrio*, *Roseburia*, *Faecalibacterium*, *Bifidobacterium*, *Ruminococcus*, *Clostridium*, *Alistipes*, and *Streptococcus*. The relative abundance data for these species are presented in Figure 2.

The characteristics at baseline in the training set

A total of 270 patients participated in the study. After random assignment, 216 patients were allocated to the training set and 54 patients to the test set. In the training set, the average age of patients was 56.68±4.81 years, with 56.54% being male. As shown in Table 1, patients with a Grade I Kellgren-Lawrence Classification after one year were younger (55.77±4.74 years) compared to those with Grade II/III (P=0.002). The BMI of patients with Grade I after one year was 25.46±5.01, which was lower than that of patients with Grade II/III (P=0.003). Additionally, the baseline WOMAC score for patients with Grade I after one year was 45.61±7.73, significantly lower than for those with Grade II/III (P<0.001). Furthermore, the abundance of *Bacteroides* and *Lactobacillus* in patients with Grade I after one year was higher compared to those with Grade II/III (P=0.004 and P=0.028, respectively).

**Table 1:** The characteristics at baseline in the training set SD, standard deviation; BMI, body mass index; WOMAC, Western Ontario and McMaster Universities Osteoarthritis Index; NSAIDs, non-steroidal anti-inflammatory drugs; FBG, fasting blood glucose; WBC, white blood cell count; CRP, C-reactive protein.

Variable	Kellgren-Lawrence Classification after 1 year		P-value
	Grade I (n=117)	Grade II/III (n=99)	
Age, years, mean ± SD	55.77±4.74	57.77±4.70	0.002
Gender, n (%)			0.431
Male	69 (58.97)	53 (53.61)	
Female	48 (41.03)	46 (46.39)	
BMI, mean±SD	25.46±5.01	27.65±5.56	0.003
WOMAC at baseline, mean±SD	45.61±7.73	49.73±8.36	
NSAIDs, n (%)			0.420
No	51 (43.59)	38 (38.14)	
Yes	66 (56.41)	61 (61.86)	
FBG, mmol/L, mean±SD	5.52±0.81	5.53±0.87	0.898
WBC, 10^9^/L, mean±SD	6.85±1.90	6.87±2.05	0.933
CRP, mg/dL, mean±SD	0.43±0.08	0.42±0.10	0.380
Knee morphology, n (%)			0.121
Normal	46 (39.32)	27 (26.80)	
Varus	44 (37.61)	48 (49.48)	
Valgum	27 (23.08)	24 (23.71)	
Bacteroides, mean±SD	0.27±0.06	0.25±0.06	0.004
Prevotella, mean±SD	0.08±0.02	0.07±0.02	0.192
Lactobacillus, mean±SD	0.03±0.01	0.03±0.01	0.028
Bilophila, mean±SD	0.02±0.02	0.02±0.02	0.528
Desulfovibrio, mean±SD	0.05±0.03	0.06±0.04	0.052
Roseburia, mean±SD	0.05±0.03	0.05±0.03	0.753
Faecalibacterium, mean±SD	0.13±0.04	0.13±0.04	0.691
Bifidobacterium, mean±SD	0.03±0.01	0.04±0.01	0.260
Ruminococcus, mean±SD	0.03±0.01	0.03±0.01	0.203
Clostridium, mean±SD	0.03±0.01	0.03±0.01	0.281
Alistipes, mean±SD	0.09±0.02	0.10±0.02	0.664
Streptococcus, mean±SD	0.06±0.01	0.07±0.01	0.153

Comparison of alpha diversity index between two groups

The Shannon index, Simpson index, and Chao1 index were calculated to evaluate the alpha diversity of patients' gut microbiota. As shown in Table 2, the Shannon index for patients with a Grade I Kellgren-Lawrence Classification after one year was lower than that for patients with Grade II/III (P=0.018). However, there were no significant differences in the Simpson index and Chao1 index between the two groups.

**Table 2:** Comparison of alpha diversity index between two groups KL, Kellgren-Lawrence Classification after one year.

Group	Shannon	Simpson	Chao1
KL: Grade I	4.655±0.159	0.850±0.038	355.83±87.69
KL: Grade II/III	4.918±0.631	0.867±0.124	364.55±187.58
P-value	0.018	0.125	0.324

Construction and validation of the prediction model

Predictors were selected using LASSO with five-fold cross-validation on the training set. The final analysis included age, BMI, WOMAC, FBG, CRP, and the gut microbiota species *Bacteroides*, *Lactobacillus*, *Bilophila*, *Desulfovibrio*, *Bifidobacterium*, *Ruminococcus*, *Clostridium*, *Alistipes*, and *Streptococcus*. A conventional risk factor model was established using age, BMI, WOMAC, FBG, and CRP. Microbiome models were developed using the gut microbiota species, while microbiome-augmented models combined all variables. Logistic regression, Random Forest, XGBoost, and LGBM were employed to build each prediction model. The performance metrics - accuracy, sensitivity, specificity, PPV, NPV, and AUC - for each model are presented in Table 3.

The best model was the microbiome-augmented model built using LGBM. In the training set, this model achieved an AUC of 0.812 (0.754-0.870), sensitivity of 0.804 (0.725-0.883), specificity of 0.744 (0.664-0.823), PPV of 0.722 (0.638-0.807), NPV of 0.821 (0.748-0.894), and accuracy of 0.771 (0.715-0.827). In the testing set, the model attained an AUC of 0.876 (0.781-0.972), sensitivity of 0.759 (0.603-0.914), specificity of 0.917 (0.806-1.000), PPV of 0.917 (0.806-1.000), NPV of 0.759 (0.603-0.914), and accuracy of 0.830 (0.729-0.931) (Table 3).

**Table 3:** The predictive effect of the model on Kellgren-Lawrence classification of patients one year later AUC, area under the curve; PPV, positive predictive value; NPV, negative predictive value; RF, Random Forest, LGBM, Light GBM; XGB, XGBoost; LR, logistic regression; RF, Random Forest.

Model	Dataset	Algorithm	AUC (95% CI)	Accuracy (95% CI)	Sensitivity (95% CI)	Specificity (95% CI)	PPV (95% CI)	NPV (95% CI)
Conventional risk factor	Train	RF	0.772 (0.710-0.835)	0.720 (0.659-0.780)	0.701 (0.610-0.792)	0.735 (0.655-0.815)	0.687 (0.596-0.778)	0.748 (0.668-0.827)
	Test		0.772 (0.647-0.896)	0.698 (0.575-0.822)	0.621 (0.444-0.797)	0.792 (0.629-0.954)	0.783 (0.614-0.951)	0.633 (0.461-0.806)
	Train	LGBM	0.745 (0.680-0.810)	0.701 (0.640-0.762)	0.732 (0.644-0.820)	0.675 (0.590-0.760)	0.651 (0.562-0.741)	0.752 (0.670-0.835)
	Test		0.749 (0.618-0.880)	0.698 (0.575-0.822)	0.724 (0.561-0.887)	0.667 (0.478-0.855)	0.724 (0.561-0.887)	0.667 (0.478-0.855)
	Train	XGB	0.751 (0.685-0.817)	0.710 (0.650-0.771)	0.691 (0.599-0.783)	0.726 (0.646-0.807)	0.677 (0.585-0.769)	0.739 (0.659-0.819)
	Test		0.756 (0.622-0.889)	0.736 (0.617-0.855)	0.586 (0.407-0.765)	0.917 (0.806-1.000)	0.895 (0.757-1.000)	0.647 (0.486-0.808)
	Train	LR	0.670 (0.598-0.742)	0.668 (0.605-0.731)	0.567 (0.468-0.666)	0.752 (0.674-0.830)	0.655 (0.553-0.756)	0.677 (0.597-0.757)
	Test		0.681 (0.533-0.829)	0.679 (0.554-0.805)	0.966 (0.899-1.000)	0.333 (0.145-0.522)	0.636 (0.494-0.779)	0.889 (0.684-1.000)
Microbiome models	Train	RF	0.812 (0.754-0.869)	0.748 (0.689-0.806)	0.784 (0.702-0.865)	0.718 (0.636-0.799)	0.697 (0.611-0.784)	0.800 (0.723-0.877)
	Test		0.878 (0.786-0.970)	0.849 (0.753-0.945)	0.931 (0.839-1.000)	0.750 (0.577-0.923)	0.818 (0.687-0.950)	0.900 (0.769-1.000)
	Train	LGBM	0.749 (0.685-0.814)	0.696 (0.635-0.758)	0.670 (0.577-0.764)	0.718 (0.636-0.799)	0.663 (0.570-0.757)	0.724 (0.643-0.805)
	Test		0.857 (0.759-0.955)	0.792 (0.683-0.902)	0.724 (0.561-0.887)	0.875 (0.743-1.000)	0.875 (0.743-1.000)	0.724 (0.561-0.887)
	Train	XGB	0.774 (0.712-0.836)	0.729 (0.669-0.789)	0.577 (0.479-0.676)	0.855 (0.791-0.919)	0.767 (0.670-0.864)	0.709 (0.634-0.784)
	Test		0.853 (0.755-0.952)	0.755 (0.639-0.871)	0.621 (0.444-0.797)	0.917 (0.806-1.000)	0.900 (0.769-1.000)	0.667 (0.506-0.828)
	Train	LR	0.635 (0.559-0.710)	0.654 (0.590-0.718)	0.608 (0.511-0.705)	0.692 (0.609-0.776)	0.621 (0.523-0.719)	0.681 (0.597-0.764)
	Test		0.710 (0.560-0.860)	0.755 (0.639-0.871)	0.690 (0.521-0.858)	0.833 (0.684-0.982)	0.833 (0.684-0.982)	0.690 (0.521-0.858)
Microbiome-augmented models	Train	RF	0.824 (0.770-0.878)	0.766 (0.710-0.823)	0.773 (0.690-0.857)	0.761 (0.683-0.838)	0.728 (0.642-0.814)	0.802 (0.728-0.876)
	Test		0.852 (0.753-0.951)	0.755 (0.639-0.871)	0.724 (0.561-0.887)	0.792 (0.629-0.954)	0.808 (0.656-0.959)	0.704 (0.531-0.876)
	Train	LGBM	0.812 (0.754-0.870)	0.771 (0.715-0.827)	0.804 (0.725-0.883)	0.744 (0.664-0.823)	0.722 (0.638-0.807)	0.821 (0.748-0.894)
	Test		0.876 (0.781-0.972)	0.830 (0.729-0.931)	0.759 (0.603-0.914)	0.917 (0.806-1.000)	0.917 (0.806-1.000)	0.759 (0.603-0.914)
	Train	XGB	0.837 (0.784-0.890)	0.776 (0.720-0.832)	0.680 (0.588-0.773)	0.855 (0.791-0.919)	0.795 (0.708-0.882)	0.763 (0.691-0.836)
	Test		0.859 (0.760-0.958)	0.792 (0.683-0.902)	0.690 (0.521-0.858)	0.917 (0.806-1.000)	0.909 (0.789-1.000)	0.710 (0.550-0.869)
	Train	LR	0.676 (0.605-0.748)	0.668 (0.605-0.731)	0.567 (0.468-0.666)	0.752 (0.674-0.830)	0.655 (0.553-0.756)	0.677 (0.597-0.757)
	Test		0.697 (0.551-0.842)	0.698 (0.575-0.822)	1.000 (1.000-1.000)	0.333 (0.145-0.522)	0.644 (0.505-0.784)	1.000 (1.000-1.000)

**Figure 3:**
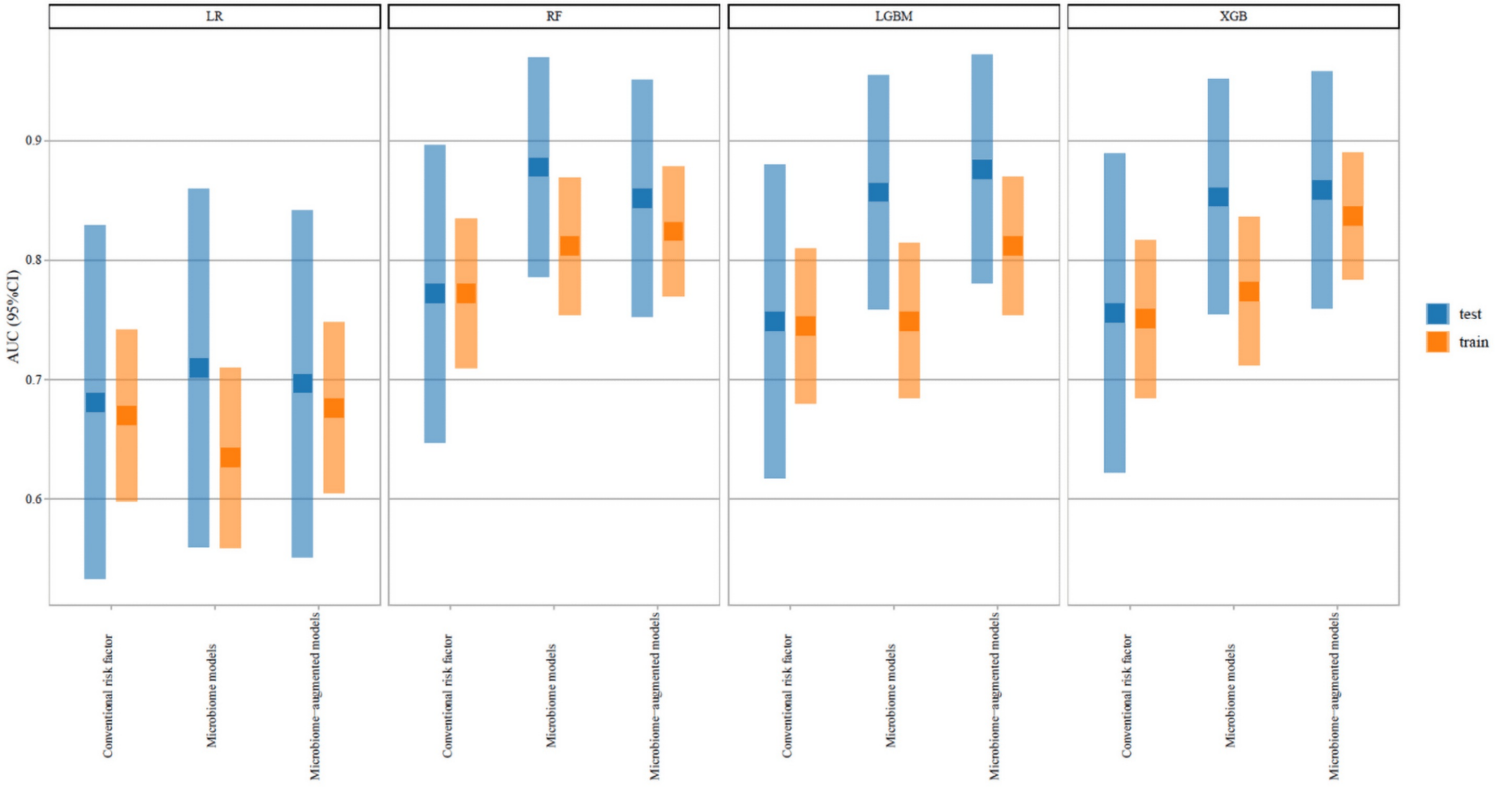
The AUCs and 95% confidence interval of 12 models AUC, area under the curve.

The AUCs for the 12 models are displayed in Figure 3. The prediction ability improved progressively from conventional risk factor models to microbiome models, and further to microbiome-augmented models. Notably, improvements were significant for the Random Forest, LGBM, and XGBoost models, with the final microbiome-augmented model demonstrating excellent prediction capability.

**Figure 4:**
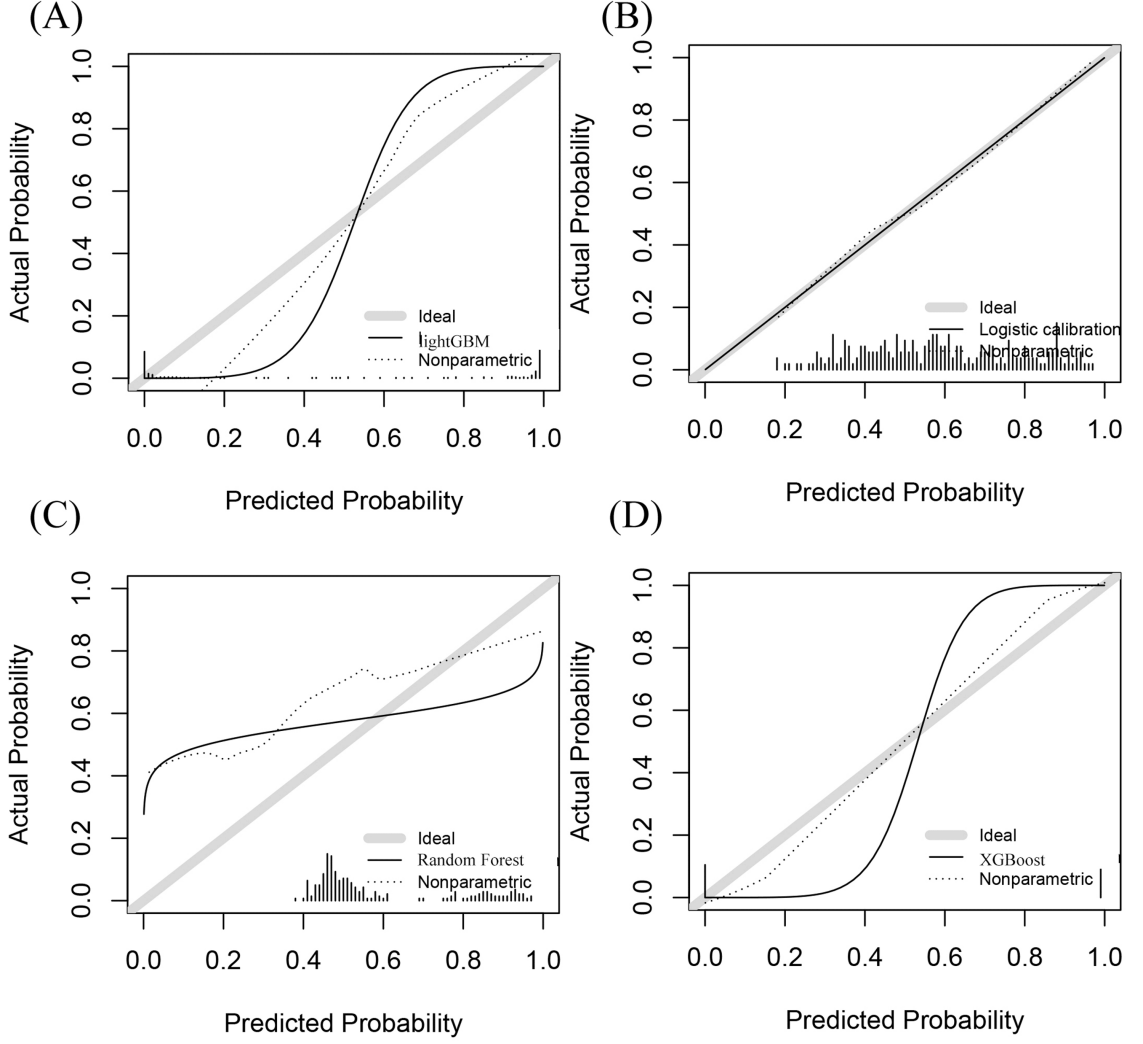
Calibration curve of microbiome-augmented models on test set (A) Calibration curve of microbiome-augmented Light GBM model; (B) calibration curve of microbiome-augmented logistic model; (C) calibration curve of microbiome-augmented Random Forest model; (D) calibration curve of microbiome-augmented XGBoost model.

**Figure 5:**
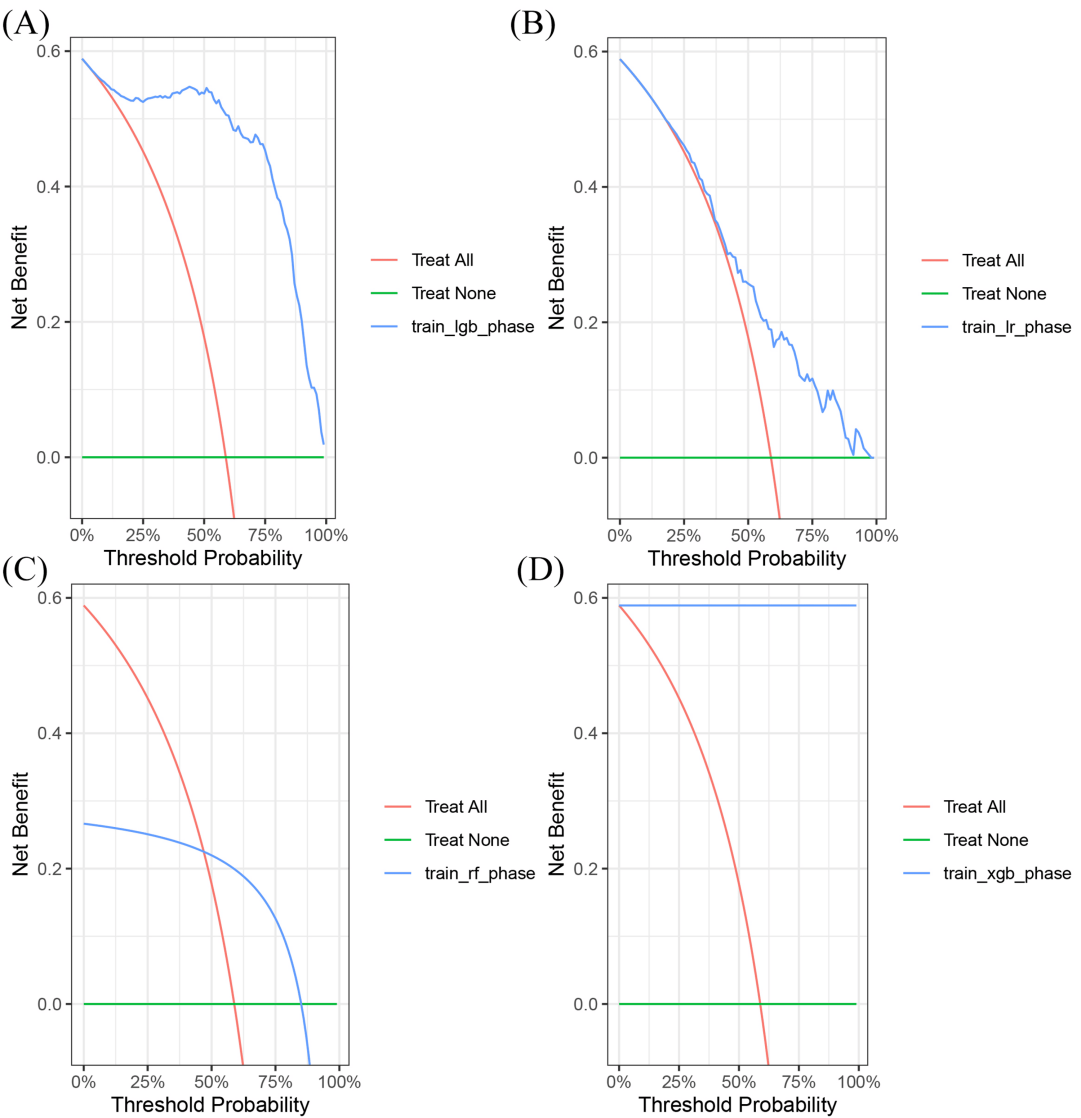
The decision curve analysis of microbiome-augmented models on the test set (A) The decision curve analysis of Light GBM on the test set; (B) the decision curve analysis of logistic on the test set; (C) the decision curve analysis of Random Forest on the test set; (D) the decision curve analysis of XGBoost on the test set.

Figure 4 and Figure 5 show calibration curves and decision curves of microbiome-augmented models on the test set, respectively. The logistic and XGBoost models showed better calibration and net benefit performance at medium thresholds compared to Light GBM and Random Forest. The parameters when developing XGBoost were set as follows: "max.depth=3", "eta=1"," nthread = 2", "nrounds=30".

**Figure 6:**
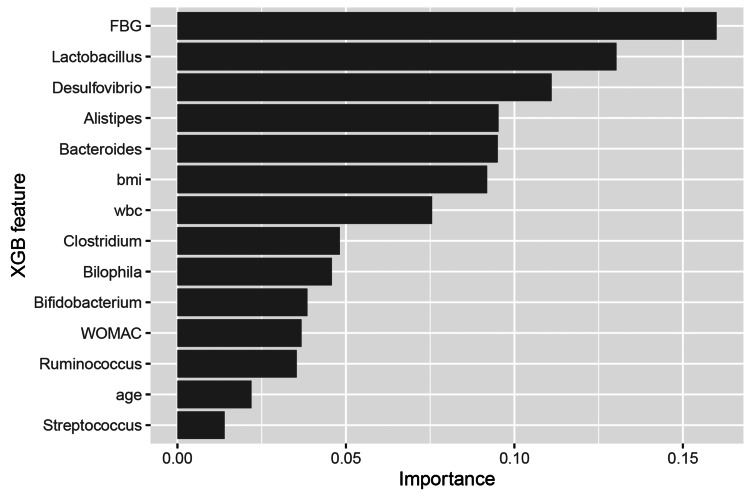
Feature importance of microbiome-augmented XGBoost FBG, fasting blood glucose; BMI, body mass index; WBC, white blood cell count; WOMAC, Western Ontario and McMaster Universities Osteoarthritis Index; XGB, XGBoost.

As shown in Figure 6, the relative importance of FBG, *Lactobacillus,* and *Desulfovibrio* were the three highest, all of which exceed 0.1. The relative importance of *Alistipes*, *Bacteroides*, BMI, and WBC were also higher than the average level (1/15).

## Discussion

Knee OA is a chronic degenerative condition that can cause joint pain, dysfunction, and even disability. Current diagnostic methods lack specificity and sensitivity, leading to challenges in early and accurate diagnosis. The gut microbiome influences OA development by modulating immune responses and inflammation which suggests the potential of using the gut microbiome as a biomarker for disease progression. This prospective cohort study included 270 knee OA patients. Their general information and gut microbiota data were collected to explore the role of the gut-joint axis in OA development and establish a prediction model for the one-year Kellgren-Lawrence classification of patients. Feature selection was performed using LASSO regression with cross-validation. The analysis included 12 species of intestinal flora. Age, BMI, WOMAC, FBG, CRP, and specific gut microbiota abundance were involved. Conventional risk factor models and microbiome models were established. Microbiome-augmented models were created by combining all variables. The LGBM model in the microbiome-augmented models showed the best performance. The models improved progressively from conventional risk factor models to microbiome models and microbiome-augmented models.

The gut microbiota can influence the progression of knee OA through various mechanisms. First, dysbiosis, an imbalance in the gut microbial composition, can lead to chronic low-grade inflammation, known as systemic inflammation, which promotes cartilage degradation, synovial inflammation, and joint tissue damage [14-16]. Second, the gut microbiota produces metabolites like short-chain fatty acids, particularly butyrate, which have anti-inflammatory properties and help maintain the integrity of the intestinal barrier. These metabolites can modulate immune cell function and reduce pro-inflammatory cytokine production, indirectly affecting the inflammatory processes involved in OA [17,18]. Third, the gut microbiota contributes to the integrity of the intestinal barrier, preventing the translocation of harmful bacteria and their byproducts into the bloodstream. Dysbiosis can compromise the gut barrier, allowing bacterial components such as lipopolysaccharides to leak into the circulation and trigger systemic inflammation, promoting joint inflammation and cartilage degradation in OA [12,19-21]. Fourth, dysbiosis can disrupt immune homeostasis, leading to an imbalance between pro-inflammatory and anti-inflammatory immune responses. This imbalance, along with alterations in regulatory T cells, can contribute to excessive inflammation in the joints, accelerating OA progression [22-24]. Finally, the gut microbiota is involved in the metabolism of dietary components, such as fiber and polyphenols, which have beneficial effects on OA by reducing inflammation, oxidative stress, and cartilage degradation. However, further research is needed to fully understand these mechanisms and explore potential therapeutic interventions targeting the gut microbiome for OA management [22,25-27].

In the current study, the microbiome-augmented models were constructed. The predictive performances were compared and the best model was selected, and the model was constructed based on the general data including age, BMI, WOMAC, FBG, and CRP, and gut microbiota data including *Bacteroides*, *Lactobacillus*, *Bilophila*, *Desulfovibrio*, *Bifidobacterium*, *Ruminococcus*, *Clostridium*, *Alistipes*, and *Streptococcus*. The model had high AUC, sensitivity, specificity, PPV, NPV, and accuracy in both the training set and the testing set, indicating a good predictive ability of the model. A prediction model for medial joint space narrowing (JSN) progression in knee OA using clustering, feature engineering, and classification algorithms was developed by Ntakolia et al. [28]. They extracted 725 features from nine categories, including medical imaging outcomes. By bounding the JSN progression of both sides of the knee, they achieved a maximum prediction accuracy of 83.3% using only 29 features, which was superior to individual knee bounding. The right knee reached a maximum accuracy of 77.7% with 88 features using a support vector machine model. The left knee had slightly better accuracy at 78.3% using logistic regression but required almost double the number of features (164). This model recognized the importance of non-imaging data such as symptoms, anthropometric data, and medical history to ensure feature heterogeneity. An MLP (multilayer perceptron)-based predictive model for knee OA onset and deterioration using data from the OA Initiative was built by Chan et al. [29]. They included six risk factor categories as input variables, but the model lacked sufficient validation. It achieved acceptable results with AUC values of 0.843 and 0.765 for knee OA onset and deterioration predictions, respectively. A multimodal machine learning model to predict the risk of knee OA progression was developed by Tiulpin et al. [30]. They used raw radiographic data as input for a deep convolutional neural network (CNN) model, which estimated the probability of knee OA progression and severity. They improved the prognosis by combining the deep CNN's prediction with non-imaging data using a GBM. This approach achieved an AUC of 0.79 and an average precision (AP) of 0.68, outperforming a reference logistic regression model with an AUC of 0.75 and AP of 0.62. Thus, the model we obtained has good predictive power on the Kellgren-Lawrence classification of patients one year later.

We provide a new non-invasive approach that eliminates the need for invasive procedures and allows for easier and more convenient monitoring of knee OA progression. By considering multiple factors, the model may provide a more comprehensive and accurate assessment of knee OA progression compared to models that rely on individual risk factors alone. Our model can also help identify patients at risk of rapid disease progression, enabling early intervention and personalized treatment strategies. In addition, it provides a new perspective for understanding the role of the gut-joint axis. There were several limitations in the present study. First, total knee arthroplasty was not used as an outcome measure for observation and prediction due to the length of follow-up. Second, external validation of the findings was not conducted. Third, the imaging data of patients were not included in the analysis because of many missing imaging data. Finally, the intestinal flora is used to predict the development of arthritis, so in the actual use of the model, there are certain requirements on the collection and processing of stool samples and the detection conditions of intestinal flora, which will limit the wide application of the model proposed in this study.

## Conclusions

In the current study, several prediction models were developed using both general patient data and gut microbiota data to predict the Kellgren-Lawrence classification of knee OA patients one year later. The best-performing model was a microbiome-augmented model constructed using the LGBM algorithm. This approach may help identify patients at risk of rapid disease progression, facilitating early intervention and personalized treatment strategies. Moreover, it offers a new perspective for understanding the role of the gut-joint axis in the progression of knee OA.
